# Wildfire national carbon accounting: how natural and anthropogenic landscape fires emissions are treated in the 2020 Australian government greenhouse gas accounts report to the UNFCCC

**DOI:** 10.1186/s13021-023-00231-3

**Published:** 2023-07-17

**Authors:** David MJS Bowman, Grant J. Williamson, Mercy Ndalila, Stephen H. Roxburgh, Shaun Suitor, Rodney J. Keenan

**Affiliations:** 1 Fire Centre, School of Natural Sciences, University of Tasmania, Hobart, TAS 7000 Australia; 2 School of Agriculture, Environment and Health Sciences, Machakos University, P.O. BOX 136, Machakos, 90100 Kenya; 3 CSIRO Environment, GPO Box 1700, Canberra, ACT r2601 Australia; 4 School of Biological Sciences, University of Tasmania, Hobart, TAS 7000 Australia; 5 School of Ecosystem and Forest Sciences, University of Melbourne, Parkville, VIC 3010 Australia

**Keywords:** Anthropogenic fires, Greenhouse gas emissions, IPCC, LULUCF, UNFCCC, Wildfire

## Abstract

Greenhouse gas (GHG) accounting of emissions from land use, land-use change, and forestry necessarily involves consideration of landscape fire. This is of particular importance for Australia given that natural and human fire is a common occurrence, and many ecosystems are adapted to fire, and require periodic burning for plant regeneration and ecological health. Landscape fire takes many forms, can be started by humans or by lightning, and can be managed or uncontrolled. We briefly review the underlying logic of greenhouse gas accounting involving landscape fire in the 2020 Australian Government GHG inventory report. The treatment of wildfire that Australia chooses to enact under the internationally agreed guidelines is based on two core assumptions (a) that effects of natural and anthropogenic fire in Australian vegetation carbon stocks are transient and they return to the pre-fire level relatively quickly, and (b) that historically and geographically anomalous wildfires in forests should be excluded from national anthropogenic emission estimates because they are beyond human control. It is now widely accepted that anthropogenic climate change is contributing to increased frequency and severity of forest fires in Australia, therefore challenging assumptions about the human agency in fire-related GHG emissions and carbon balance. Currently, the national inventory focuses on forest fires; we suggest national greenhouse gas accounting needs to provide a more detailed reporting of vegetation fires including: (a) more detailed mapping of fire severity patterns; (b) more comprehensive emission factors; (c) better growth and recovery models from different vegetation types; (d) improved understanding how fires of different severities affect carbon stocks; and (e) improved analysis of the human agency behind the causes of emissions, including ignition types and fire-weather conditions. This more comprehensive accounting of carbon emissions would provide greater incentives to improve fire management practices that reduce the frequency, severity, and extent of uncontrolled landscape fires.

## Background

Annual global carbon budgets must integrate various data streams and model estimates of carbon dynamics involving emissions from fossil fuel sources, land use changes and atmospheric, oceanic, and terrestrial sinks [1]. Unquestionably, management of terrestrial biosphere carbon stores is pivotal for achieving climate policy objectives [2], and hence the United Nations Framework Convention on Climate Change (UNFCCC) has specific requirements for reporting of emissions from land use, land-use change and forestry (LULUCF) [3]. Comparatively speaking, greenhouse gas (GHG) emissions from fossil fuel sources are much easier to track and control than LULUCF because the former mostly involve irreversible, industrial processes, whereas LULUCF involves a highly dynamic interplay between anthropogenic and Earth systems processes [4]. Accounting for emissions and removals due to landscape fires is a complex aspect of LULUCF, as these disturbances can be considered as either natural, anthropogenic, or a complex mix of both [5–7]. An additional complication is that fire also involves emissions of multiple GHGs (e.g. carbon dioxide, methane and nitrous oxide) and particulates [8]. Accounting for landscape fire is particularly pertinent for Australia, given the prime importance of fire in shaping the ecology of the continent [9], the size of the land mass relative to population, as well as the nation’s historical reliance of LULUCF activities to meet international emission reduction targets [10].

The recent paper by Ndalila et al. [11] touched on the complexity of accounting for forest fires in Australia using a case study of a very intense wildfire in the Australian state of Tasmania in 2013. This paper applied  a bottom-up method for estimating carbon dioxide and particulate emissions from landscape fires. This method was subsequently applied to estimate GHG emissions from the 2019-20 forest fires along Australian eastern seaboard [12], with results found to be in close agreement with an independent and more sophisticated method involving atmospheric chemistry and remote sensing [13]. The substantial emissions from the 2013 wildfire prompted Ndalila et al. [11] to discuss the role of landscape fire in recent Tasmanian GHG accounts [14]. These accounts suggested the Tasmania had achieved carbon neutrality, primarily due to its high reliance on hydro-electric power, reductions in emissions from harvesting in mature forest and carbon sequestered in regrowing forest. Unfortunately, those authors incorrectly stated that the combustion of logging debris is not included in annual estimates of bushfire emissions. Here, we expand the Ndalila et al. [11] discussion of Australian carbon accounting of landscape fire, to correct misapprehensions about accounting of emissions from the forestry sector and to propose improved accounting approaches relating to landscape fire.

## Main text

### Australian GHG accounting

Australian reporting of GHG emissions was initiated in the Kyoto Protocol (KP) which required Parties with reporting obligations to include net emissions from a specific subset of land sector classes and activities [15], noting that the KP has been superseded by the Paris Agreement in 2020. Inventory submissions including LULUCF have been undertaken under the UN Framework Convention on Climate Change since 2003 [16]. As part of the submission to UNFCCC, the Australian Government currrently provides detailed estimates of GHG emissions from the National Greenhouse Gas Inventory (NGGI), including from the Land Use Land Use Change and Forestry (LULUCF) sector [17, 18].

Following guidelines developed by the Intergovernmental Panel on Climate Change (IPCC) and required by the UNFCCC [19], Australian reporting of net LULUCF emissions is broken down into six land categories: forest lands; cropland; grassland; wetland; settlements; and other lands; with further subdivisions amongst categories based on geographic domain (e.g. temperate, subtropical and tropical), and the type of land use (e.g. forestry, grazing, cropping) and land use change [17]. All land categories are assumed to be under human management except ‘other lands’ (broadly defined as arid unproductive lands in central Australia). The ‘Other lands’ category is excluded from the NGGI even though some are burned by pastoralists and Indigenous people to manage vegetation [20]. Although Australia’s GHG accounting methods are explicitly based on and compliant with the rules in the UNFCCC guidelines [19], data and models used to generate the GHG estimates are nationally determined and customised in accordance with good practice for inventories established by the IPCC [15, 17, 21]. The Australian approach to fire emissions is not widely understood in the scientific community [17]. Below we explain the methods for estimating Australian landscape fire emissions, particularly focusing on the differentiation of ‘natural’ and ‘managed’ forest fires.

### Emission estimates from LULUCF

**Fig. 1:**
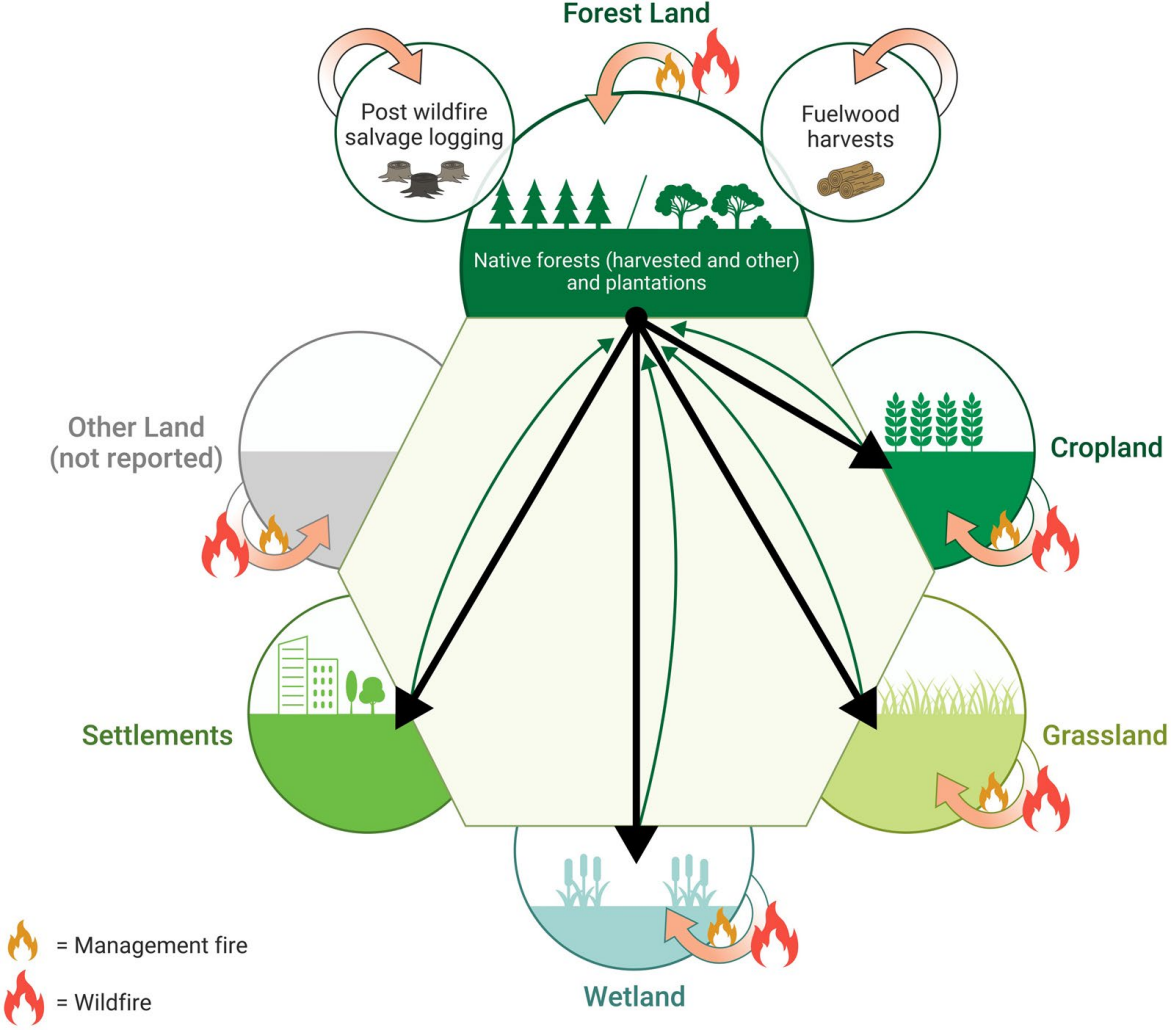
Conceptual model of how landscape fire is represented in Australian Government greenhouse gas accounts reported to the UNFCCC. The report presents GHG emissions and removals across five land categories (Forest Land, Cropland, Grassland, Wetland, Settlements that are classified as being under anthropogenic management). It excludes unproductive, arid Other Lands category. Fire is understood to occur in all fire management lands. It is assumed carbon emissions are balanced by regrowth over time, albeit varying spatially, temporally, and among vegetation types. The report also includes consideration of wood harvests, post-fire salvage logging, and fuelwood collection in native and non-native plantations. Fire can be used in activities that convert one land category to another category (resulting in carbon losses, black arrows), for example clearing of forest, noting some of these deforestation emissions can be sequestered by afforestation or reforestation (green arrows)

As indicated above, Australia’s national greenhouse gas inventory (NGGI) for the land sector is based around five land cover/use categories: the unproductive arid Other Lands category is excluded (Fig. 1). Landscape fire is understood to occur in all land cover categories except urban areas. It is assumed carbon emissions from landscape fires are balanced by regrowth over time, albeit varying spatially, temporally, and among vegetation types. The NGGI also includes consideration of wood harvests, post-fire salvage logging, and fuelwood collection in native and non-native plantations. Emissions estimates in all categories are based on integration of the best available data from various streams, including national scale vegetation type maps; fine scale remote sensing analyses of land cover change (Landsat 25 m pixels); and mechanistic modelling of landscape carbon dynamics, including soil carbon, for different vegetation types driven by interpolated national climate statistics [21]. Data are included from some Australian states on timber harvesting and forest regeneration burning, and prescribed burning extent for all jurisdictions except the Northern Territory, where all fires are reported as savanna fires and not differentiated by wildfire or prescribed burning [22].

### Fire emission assumptions

The NGGI is based on four key assumptions about landscape fire emissions [17]. First, fire disturbance is considered to have a transient effect on GHG emissions because post-fire vegetative recovery mechanisms are extremely well-developed in most Australian flora. Second, and relatedly, estimates of GHG emissions from fires used to regenerate logged forests and fires used to reduce fuel loads in forests and grassland, or to manage pasture and cropland, are calculated in different ways depending on the nature of the fire. For certain types of fire, effects are considered short-lived with emissions balanced by carbon uptake in recovering vegetation within a few months or up to 10 to 20 years, depending on the vegetation type and fire intensity. Emissions associated with conversion of forest to other land categories are modelled using parameters that consider both immediate losses due to fire and decay of woody material and emissions of soil carbon over time. Removals associated with conversion from non-forest to forest classes through reforestation and afforestation are also modelled [17]. Third, managed landscape fires are assumed to be less severe and patchier than unmanaged landscape fires. Finally, some unmanaged landscape fires are disaggregated from Australia’s national totals and reported separately in the National Inventory Report. Recognising that national greenhouse gas inventories are designed to reflect human-induced emissions, the IPCC has agreed on a set of guidelines to identify and separately report emissions from non-anthropogenic fires, commonly referred to as a ‘natural disturbance provision’, with Australia using a modelling approach to provide evidence that emissions and subsequent removals from natural disturbances ‘average out’ over time [23]. In this process, the area of forest subject to a natural disturbance is disaggregated, tracked, and reported on separately from the point in time it was disturbed until the time forest carbon has returned to its original state, or it is identified that the forest has failed to regrow. If the forest is deemed to have failed to regrow following a fire event, this will be reflected in the time series as a forest conversion and counted as an anthropogenic emission in the National Inventory. The rationale for this natural disturbance provision [7, 19] is to meet the primary purpose of NGGIs to report on anthropogenic emissions and removals. Emissions resulting from extreme fire weather conditions are considered beyond human control and therefore not anthropogenic. Parties subject to such events may struggle to accurately represent actual changes and trends in anthropogenic emissions and removals. This can result in over- or under-estimation of parties’ progress towards their commitments, depending on the specifics of the commitment period and national circumstances.

### Differentiating natural (unmanaged) and managed forest fires

In the NGGI, these managed (anthropogenic) fires and unmanaged (‘natural’) fires are differentiated using a statistical approach, with a threshold of fire scale determined for a baseline period, beyond which fires are deemed to be natural. Operationally this involves two steps:

1. A baseline of annual gross emissions from fires from 2000 to 2012 is used to determine the ‘normal’ variation in fire season emissions and to classify the scale of future fire seasons. If emissions during a subsequent fire season are more than two standard deviations beyond the baseline mean, the season is classified as anomalous.
2. Each anomalous fire season is further investigated to determine if the area burned in each state and territory exceeds by one standard deviation the average area burned during the 2000–2012 baseline. Reported extent of prescribed burning is excluded from this analysis. If this threshold is exceeded then all fire related GHG emissions (excluding prescribed fires) from that state or territory are classified as originating from unmanaged (‘natural’) fires [17] and reported as such in the national greenhouse gas accounts. The parameters used this assessment are provided in Table 1.

**Table 1:** Thresholds and number of ‘natural disturbances’ caused by unmanaged forest fires in Australian States and Territories between 1990–2020 in the Australian National Greenhouse Gas Inventory [17]

	Geographic UNIT	Units	Statistic based on 2000–2012 baseline)	Threshold value	Number natural disturbance years
Step 1 Gross GHG emissions	National	CO^2^-e kt	µ + 2 σ	71,950	6
Step 2 Area burned by fire	Australian Capital Territory (ACT)	kha	µ + σ	0.01	3
	New South Wales (NSW)	kha	µ + σ	223.92	3
	Queensland (QLD)	kha	µ + σ	167.94	2
	South Australia (SA)	kha	µ + σ	42.40	3
	Tasmania (TAS)	kha	µ + σ	16.77	4
	Victoria (VIC)	kha	µ + σ	122.01	5
	Western Australia (WA)	kha	µ + σ	348.36	4

Estimation involves a two-step process: estimation of nationally anomalous (> mean (µ) plus two standard deviations (σ)) GHG emissions for a given year relative to 2002–2012 reference period (Step 1). If a given year is considered anomalous in Step 1, then the area burned in each State and Territory is estimated, to determine if this exceeds the threshold by one standard deviation of the non-natural disturbance years (Step 2). This only applies to non-natural disturbance years. Years with natural disturbances for a given state are excluded from the calculation. Emission from ‘natural disturbance years’ are deemed *force majeure* and not reported as human-induced emissions in the GHG accounts of each State or Territory. Northern Territory is excluded from the NGGI assessment because the ‘national definition of natural disturbances applies to wildfires in temperate forests, and does not apply to fires reported as controlled burning (e.g. in temperate forests or in wet-dry tropical forests and woodlands)’ [17]

It is important to note that the distinction between managed and unmanaged fires is acknowledged in NGGI as being blurred because some of the deemed ‘anthropogenic’ emissions are also likely to be emitted from natural fires as well anthropogenic fires’ [24]. To control for this remaining inter-annual variability, the long-run trend in carbon stocks is reported, reflecting the balance of the carbon lost in the fire and that re-absorbed by regrowth.

## Discussion

### Fire and GHG accounting

**Fig. 2:**
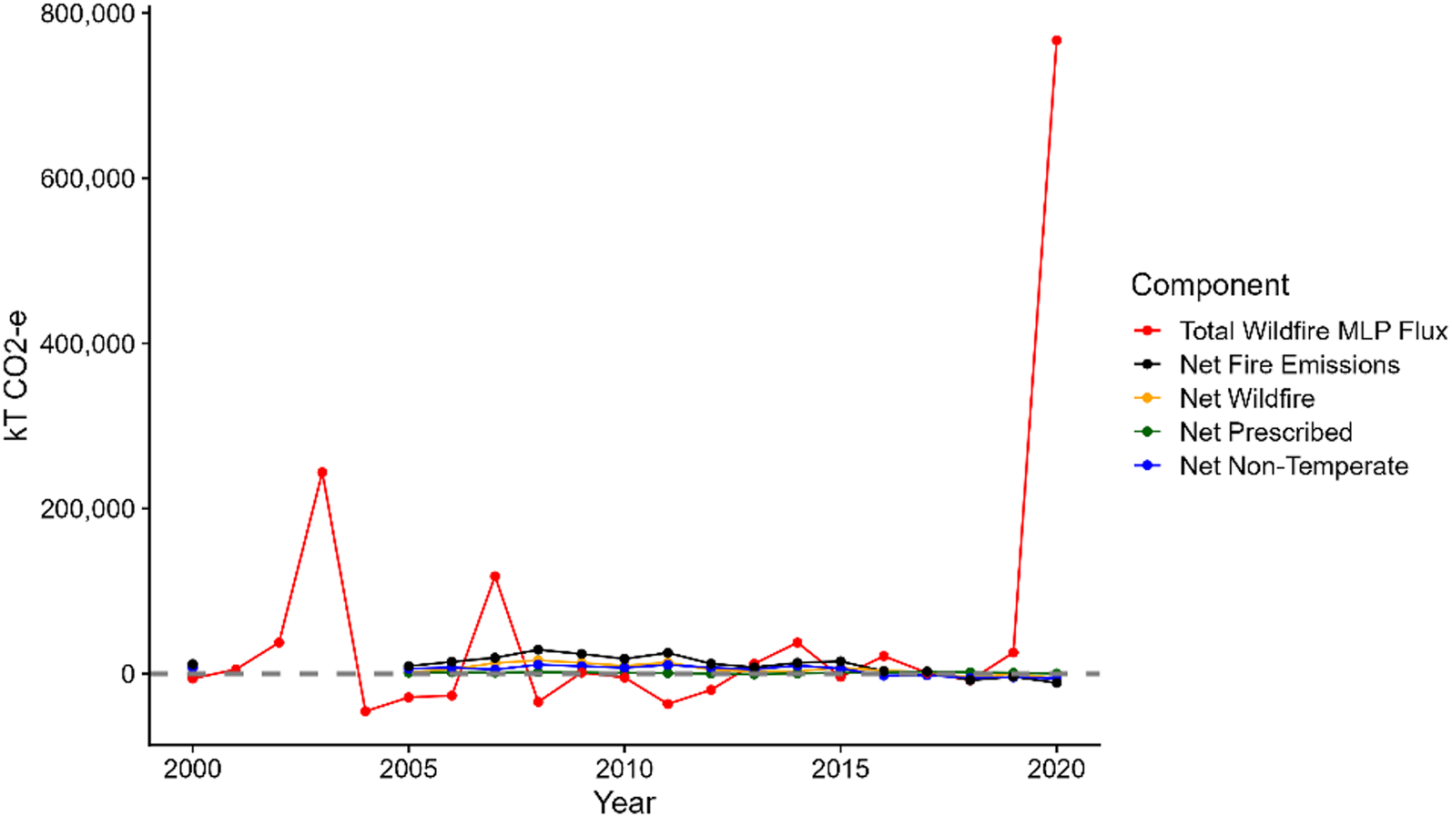
Total greenhouse gas flux (in CO_2_-e) from wildfires on managed forest lands between 2000–2020 (red line). Also shown are the estimated net greenhouse gas emissions (in CO_2_-e) according the Australian Government report to the UNFCCC [17] for all fires in forests (black line) broken down by temperate wildfires (yellow), temperate prescribed fires (green) and non-temperate fires (blue)

Including emissions from landscape fires in national GHG emission accounting demands a pragmatic and practical interpretation of current IPCC guidelines [19], given the constraints of available data, the complexities of the processes involved and current computational capacities. The 2022 Australian submission to the UNFCCC, in which emissions from the 2019-20 fire season were included, involved separate tracking and reporting of emissions and subsequent removals from the 2019-20 bushfires, concentrated in *Eucalyptus* forests within the state of NSW [12], because they are statistically anomalous and considered unmanaged [25] (Fig. 2). Emissions from these fires have been estimated to be 1.65 (715 vs. 433 Tg CO_2_) times the total national anthropogenic CO_2_ emissions for all economic sectors in 2019 [26]. However, looking ahead, the increase in frequency of extreme fire seasons, coupled with greater and more comprehensive understanding of their ecological effects, creates the need for review of the current approach. The key issue is whether we can continue to assume that carbon stocks in large areas of intensively burnt Australian forests can rapidly recover to their pre-fire levels.

### Climate change, forest fire and carbon balance

It is unclear whether depletion of carbon stocks from the 2019-20 Australian wilfires, driven by climate change [27], will be completely restored. Therefore, IPCC rules for reporting anthropogenic fire-related emissions are being challenged by this uncertainty. For example, remote sensing studies suggest the very large emissions from the 2019-20 fire (combined with the extended drought in the period preceding the fires) are being offset by rapid post fire regrowth enhanced by abnormally high rainfall following the fires [28], but it is unclear whether landscape carbon stocks will be completely restored. The extraordinary scale and intensity of the fires [25], has prompted speculation that these systems may switch from substantial sinks to sources of carbon [12, 13].

Such extreme fires challenge the assumption that landscape fire emissions rapidly recover to previous levels for all land categories, particularly for forests [17]. For instance, recent high frequencies of fire activity have caused areas of obligate seeding *Eucalyptus* forests to suffer population collapse after multiple fires, because regrowing forests from previous fires had not matured sufficiently to set seed, and no seed was in burnt tree crowns to provide for regeneration [29]. Recent evidence also indicates that resprouter forests, typically of drier areas that usually recover rapidly after fire, may also vulnerable to such collapse due to the increasing frequency and severity of fires [30]. Additionally, extreme drought conditions have affected other vegetation types, such as rainforest and riverine forests, that are typically too moist to burn [31, 32]. This will impact on forest carbon stocks because these ecosystems are poorly adapted to fire and their recovery is very slow. In a nutshell, climate change appears to be causing more frequent and severe fires in a broad cross section of forest types, affecting their ability to recover and restore carbon stocks. The increase in megafires in southern Australian eucalypt forests over the last 20 years raises questions about the assumption of ‘average’ fire conditions in these forests and whether this average is likely to rapidly change. The question for policy consideration is whether humans have agency to affect the emissions associated with these fires and can facilitate the recovery of carbon stocks.

### Monitoring fire regime effects on carbon balance

Climate change is causing fire regimes to shift outside the range of historical variability, thereby affecting long-term landscape carbon balances. Climate change-induced biomass loss from anomalous fires are captured, to some degree, by the Australian LULUCF national GHG inventory accounting because satellite monitoring of burned vegetation is used to determine any vegetation type conversion [17]. However, explicitly incorporating satellite monitoring of fire severity into GHG accounting methods would substantially improve estimates of fire-related emissions because it could build on observed relationships between fire severity, tree mortality and changes in carbon stocks [33]. The Australian Government already uses a seasonal proxy (early and late dry season fires) for fire severity to estimate methane and nitrous oxide emissions in the savanna burning methodology widely used to generate carbon credits (ACCUs) in the Australian monsoon tropics [34]. This methodology supports trading of emissions reductions by rewarding demonstrable reductions in the areal extent of late dry season fires, which burn at high severity and generate higher methane and nitrous oxide emissions. Burning in mosaic patterns in the early dry season results in lower severity fires likely to increase carbon storage in the landscape.

We suggest that national greenhouse gas accounting in Australia considers how the frequency and extent of fires with different severities affects the carbon balance of all vegetation types across the continent. While the Landgate AVHRR fire mapping provides a basis for more consistent monitoring and recording of fire activity across Australian states and territories, a national fire monitoring facility would provide a platform for improved assessment and reporting of fire impacts, including fire severity [25].

Further research and development into emissions of CO_2_ and other GHGs from landscape fires in different vegetation types and management regimes across Australia would also improve capacity to comprehensively report on fire-related emissions. For instance, a specific methodology has been developed to estimate non-CO_2_ GHG emission from tropical savanna carbon management programs [34], and such an approach could be developed for other vegetation types. Further, currently, there is a marked mismatch between estimates of GHG emissions from managed and unmanaged forest fires and the far greater level of detail used to report on emissions from production forests, with the latter including estimates of emissions from combustion of forest debris and the effect of fire on soil carbon (Fig. 2) [17], a fact that Ndalila et al. [11], incorrectly suggested was not the case.

A key step in setting research priorities to understand fire regime effects on forest GHG emissions, is an explicit, transparent explanation of the current methods used by the Australian Government to quantify these effects. Currently, these methodologies and approaches are described in a variety of publications and government reports, so it is difficult to form a comprehensive picture and understand the operational details.

### The challenge of attributing anthropogenic fire effects on carbon stocks

The UNFCCC and related agreements focus on reducing anthropogenic impacts on the global climate system. Distinguishing anthropogenic from ‘natural’ effects on the determinants of wildfire emissions (that is, fire extent and severity) are actively debated. For example, a key feature of recent fires in southern Australia is the increase in ignitions associated with dry lightning storms [27, 35] contributing to the dramatic rise in pyroCB events, in which uncontrolled fires create their own weather conditions and lightning ignitions [36]. Some researchers have suggested that such extreme fire behaviour has been exacerbated by past native forest logging [37], while detailed analytical evidence indicates fire extent and severity are driven primarily by climate, fire weather and landscape factors such as topography, and that the impact of past logging is relatively small and variable [38]. Other researchers posit that the fire extent and intensity have been exacerbated by cessation of Indigenous fire management in southern Australia, and that restoring this management can reduce catastrophic fires [39].

These contrasting perspectives present substantial challenges to the comparatively simple classification of unmanaged (‘natural’) and managed (anthropogenic) fires used in the current Australian NGGI [7, 17]. We suggest at the very least, with improved fire monitoring and mapping, it would be possible to differentiate fires ignited by lightning and those from anthropogenic sources [25]. Such a classification would help in differentiating whether emissions from fires can be attributed to natural or anthropogenic causes, noting this simple binary based on ignitions ignores whether the fires subsequently exceeded management agency capacity to control them. In this context, we are also putting to one side, thorny, unresolved, and more philosophical, questions of the indirect effects of anthropogenic climate change, cessation of Indigenous fire management and legacies of land management practices, all of which have probably played some role in exacerbating fire risk. Clearly, a major research challenge is determining the contermorary ‘counter-factual’ of wildfire activity that can be used to understand the effectiveness of fire and fuel management.

### Fire management and carbon stocks

Maintenance of terrestrial carbon stores under rapid anthropogenic climate change demands a spectrum of landscape interventions, such as prescribed burning, forest thinning, and cutting of fire breaks as well as fire suppression measures to mitigate the effects of dangerous, uncontrollable fires. Ndalila et al. [11] have argued that excluding GHG emissions from statistically anomalously large wildfires from national accounts could disincentivise investment in fire and forest management to protect carbon stocks, reduce fire emissions and improve resilience and recovery from fire, because there is no national carbon ‘penalty’ associated with these massive uncontrolled bushfires. We suggest that accurate accounting of all managed and unmanaged landscape fires in NGGI would provide objective evidence to motivate more government investment in forest and fire management, which is essential to protect and enhance terrestrial carbon stocks in a rapidly changing climate [25]. The savanna burning programs provide an example of how fire management has been incentivized through government greenhouse gas management programs [34].

## Conclusions

Inclusion of landscape fire in national carbon accounts is a critical but complex challenge. The Australian approach facilitates separate tracking and reporting of emissions and removals from fire seasons with statistically anomalous large, burned forest areas, because they are deemed ‘natural’, consistent with IPCC guidelines. However, increasing evidence suggests fire frequency and intensity are increasing due to anthropogenic climate change. To more accurately understand how landscape fires affect emissions from Australia’s forests demands improved understanding of the effects of fire frequency, extent and severity on landscape carbon dynamics for a range of different vegetation types. Detailed analytical methods currently used by the Australian Government to assess carbon dynamics in harvested temperate forests, and those used to assess GHG emissions from tropical savanna burning programs, could be adapted for other vegetation types. This would provide more accurate estimates of emissions from landscape fires across the entire continent. Greater investment in fire severity mapping and better detection of fires ignited by lightning are also essential to broadly differentiate emissions from unmanaged (‘natural) and managed (‘anthropogenic’) fires, although more research is required to more precisely separate natural and anthropogenic fires. Using these techniques would improve national carbon accounting for the land sector and provide greater incentive for improved forest and fire management to avoid emissions and support more rapid recovery from massive landscape fires.

## Data Availability

Not applicable.

## Abbreviations

GHG: Greenhouse gas
IPCC: Intergovernmental Panel on Climate Change
KP: Kyoto Protocol
NGGI: National Greenhouse Gas Inventory
LULUCF: Land use, land-use change and forestry
UNFCCC: United Nations Framework Convention on Climate Change
